# Enhancing the Interfacial Shear Strength and Tensile Strength of Carbon Fibers through Chemical Grafting of Chitosan and Carbon Nanotubes

**DOI:** 10.3390/polym15092147

**Published:** 2023-04-30

**Authors:** Jingyue Xiao, Huigai Li, Munan Lu, Yuqiong Wang, Jin Jiang, Wengang Yang, Shuxuan Qu, Weibang Lu

**Affiliations:** 1 School of Nano-Tech and Nano-Bionics, University of Science and Technology of China, Hefei 230026, China; 2 Division of Advanced Nanomaterials and Innovation Center for Advanced Nanocomposites, Suzhou Institute of Nano-Tech and Nano-Bionics, Chinese Academy of Sciences, Suzhou 215123, China; 3 College of Textile and Clothing Engineering, Soochow University, Suzhou 215021, China; 4 Division of Nanomaterials and Jiangxi Key Lab of Carbonene Materials, Jiangxi Institute of Nanotechnology, Nanchang 330200, China

**Keywords:** carbon fiber, chitosan, carbon nanotubes, chemical grafting, interfacial shear strength

## Abstract

Multi-scale “rigid-soft” material coating has been an effective strategy for enhancing the interfacial shear strength (IFSS) of carbon fibers (CFs), which is one of the key themes in composite research. In this study, a soft material, chitosan (CS), and a rigid material, carbon nanotubes (CNTs), were sequentially grafted onto the CFs surface by a two-step amination reaction. The construction of the “rigid-soft” structure significantly increased the roughness and activity of the CFs surface, which improved the mechanical interlocking and chemical bonding between the CFs and resin. The interfacial shear strength (IFSS) of the CS- and CNT-modified CFs composites increased by 186.9% to 123.65 MPa compared to the desized fibers. In addition, the tensile strength of the modified CFs was also enhanced by 26.79% after coating with CS and CNTs. This strategy of establishing a “rigid-soft” gradient modulus interfacial layer with simple and non-destructive operation provides a valuable reference for obtaining high-performance CFs composites.

## 1. Introduction

In pursuit of carbon neutrality, developing lighter and stronger structural materials has long been considered one of the key themes in industries such as aerospace, land/air transport, and civil infrastructures. Currently, among the most commonly used engineering materials, carbon fibers reinforced polymeric composites (CFRPs) possess the highest specific strength and modulus, making them ideal materials for constructing lightweight structures [1,2]. It is well known that the overall performance of CFRPs is not only determined by the intrinsic properties of carbon fibers (CFs) and matrix materials [3,4] but also greatly affected by the interface between these two compounds. However, the surface of CFs was inert and almost free of active groups, which caused weak interface bonding with the matrix since CFs were made through high-temperature processing [5,6,7]. To improve the interfacial adhesion between CFs and resin, the surface of CFs are modified by many methods, such as the application of acidic oxidation [8,9], electrochemical oxidation [10,11,12], chemical vapor deposition (CVD) [13,14,15,16], high-energy radiation [17,18], and plasma treatment [19]. However, the application of these methods is limited by the deterioration of the mechanical properties of the fibers, high energy consumption, and environmental unfriendliness [20,21]. Therefore, it is necessary to develop a simple and non-destructive method to modify the surface of CFs.

Many materials are used to construct interfacial layers to enhance the interfacial strength between carbon fibers and resin matrix and they are categorized into two groups, soft materials and rigid nanomaterials. Flexible polymers with reactive functional groups such as polydopamine (PDA) [22,23], polyether amine (PEA) [24,25], and poly(amido amine) (PAMAM) [26,27] are referred to as soft materials. Nanomaterials with higher rigidity such as graphene oxide (GO) [28,29,30], halloysite nanotubes (HNTs) [31], carbon nanotubes (CNTs) [32,33,34], and graphitic carbon nitride (C_3_N_4_) [35] are called rigid nanomaterials. The surface of the soft material is rich in functional groups and has good flexibility, which can effectively enhance the chemical bonding with the resin matrix and relax the interfacial stress [24,36,37]. Since the modulus of polymers is much lower than that of CFs, optimal modulus matching has still not been achieved [38,39]. The stiffened modulus intermediate layer formed by the rigid nanomaterial not only increases the CFs’ surface roughness but also shields the crack propagation from touching the fiber surface [13,40,41]. However, in most cases, rigid nanomaterials tend to aggregate due to their incompatibility with the polymer matrix, leading to stress concentrations and degradation of composite properties [42].

Researchers have attempted to create “rigid-soft” interfacial layers to enhance the bond between the fibers and the resin matrix, considering the advantages of soft and rigid interfacial layers. Interface layers have been formed by PAMAM and CNTs [43,44], PEA and GO [45], PEA and CNTs [46,47], PDA and nano-silica (SiO_2_) [48,49], and PDA and CNTs [15,50]. For example, Pu et al. [51] assembled positively charged polyethyleneimine (PEI) and negatively charged nanocomplexes composed of a metal–organic backbone (MOF) and graphene oxide (GO) onto carbon fibers (CFs) via electrostatic interactions. This treatment led to an increase in polar groups, wettability, and surface roughness on the surface, which increase the IFSS by 42.12% compared to the original CFs. However, the construction of a “rigid-soft” structured interfacial layer relying only on electrostatic interactions has limited improvement in the interfacial properties of the composite. Zheng et al. [46] constructed CF-CNT-PEA “rigid-soft” multi-scale gradient modulus interlayers in CFs by establishing π-bridges between CFs and CNTs with the assistance of pyrene compounds and then chemically grafting polyetheramine (PEA) onto the CF surface. The IFSS of CNT-PEA/epoxy resin (EP) was improved by 71.1% compared to the untreated resin. As the CNTs and CFs were connected only by a weak π–π interaction, the CNTs tend to fall off from the fiber surface during processing, which may degrade the composite properties. It is necessary to develop a simple and effective method of rigid and flexible interfacial layers firmly fixed on the CFs surface.

In this work, a new “rigid-soft” interfacial layer structure was constructed using CS and CNTs by a two-step amination reaction. At room temperature, CS was chemically grafted to the CFs surface as the “soft” phase, and then the “rigid” CNTs were chemically grafted to the CF@CS fiber surface to improve the interfacial strength. This approach resulted in a more firm grafting of CS and CNTs onto the CFs surface, and the chemical bonding improved the interfacial strength more effectively. The interfacial properties of the CF@CS@CNT/EP at different CNT concentrations were systematically investigated through IFSS testing. A reasonable reinforcement mechanism was proposed to illustrate the synergistic enhancement of the “rigid-soft” structure. Meanwhile, the effect of this approach on the tensile behavior of CFs was evaluated by monofilament tensile tests. This novel “rigid-soft” interface, which was non-destructive and robust, can be used as a design guide for the development of composites with improved strength and toughness by incorporating nanomaterials and locally deformable materials with low stiffness.

## 2. Experimental

### 2.1. Materials

Polyacrylonitrile-based carbon fiber (T700,12K, Toray, Tokyo, Japan) with an average diameter of 7 μm was supplied by Shenzhen Carbonene Technology Co., Ltd. (Beijing, China) The as-received carbon fibers (AR-CF) were coated with sizing materials, while the chemicals of the sizing materials were not removed by the supplier. Single-wall CNTs (TUBALL^TM^, diameter 3–5 nm, length > 5 μm) were supplied by OCSiAl, Russia. Additionally, 2-(7-Azabenzotriazol-1-yl)-N,N,N′,N′-tetramethyluronium hexafluorophosphate (HATU), glacial acetic acid, K_2_S_2_O_8_, AgNO_3_, and chitosan (CS, 95% deacetylated, viscosity 100–200 mPa s) were purchased from Shanghai Maclean Biochemical Technology Co., Ltd. (Shanghai, China). N,N-Dimethylformamide (DMF) was supplied by China National Pharmaceutical Group Chemical Reagent Co., Ltd. (Beijing, China). Epoxy resin (TED-85) was supplied by Guangzhou Tiantai High-Tech Co., Ltd. (Guangzhou, China), and the curing agent was homemade in the laboratory.

### 2.2. Surface Treatment of CNTs and CFs

**Desizing of CFs.** As the details of the sizing materials on the as-received CFs surfaces were unknown, the as-received CFs were first desized by using the Soxhlet extractor method [52], in which CFs were refluxed in acetone at 85 °C for 48 h, washed by using deionized water, and finally dried overnight at 80 °C. The obtained fibers were referred to as desized CF.

**Functionalization of CFs and CNTs.** The desized CFs were oxidized at 70 °C for 1 h in a mixture of AgNO_3_ (0.01 mol/L) and K_2_S_2_O_8_ (0.1 mol/L) [53], and the obtained oxidized CFs were referred to as CF-COOH. To functionalize CNTs, 0.1 g of CNTs was mixed with 100 mL sulfuric acid/nitric acid (3/1) solution at 60 °C for 4 h [54], and the mixture was then neutralized by using deionized water. The carboxylated CNTs were collected through vacuum-assisted filtration, washed with deionized water, and dried in an oven at 60 °C for 24 h.

**Grafting of CS and CNTs onto CFs surfaces.** To chemically graft CS onto CFs, 0.1 g CF-COOH, 0.06 g triethylamine, and 0.06 g HATU were added into a 100 mL CS/DMF solution with a CS concentration of 0.1 wt.%, and then we stirred the mixture at room temperature for 18 h. The fibers were removed and then washed by using a 2% glacial acetic acid solution to remove the incompletely reacted CS, followed by drying in an oven at 60 °C for 24 h. The obtained fibers were referred to as CF@CS. To further chemically graft CNTs onto CF@CS, a series of CNTs dispersions, where the concentrations of CNTs were set as 0.025 wt.%, 0.05 wt.%, 0.1 wt.%, and 0.15 wt.%, were first prepared by adding a certain amount of carboxylated CNTs into 100 mL DMF, and then the mixtures were sonicated for 2 h. Then, 0.1 g of CF@CS, 0.06 g of triethylamine, and 0.06 g of HATU were added into 100 mL of different CNTs dispersions, and then the mixture was stirred at room temperature for 18 h. The fibers were collected and dried at 80 °C for 24 h. The obtained fibers were denoted as CF@CS@CNT1, CF@CS@CNT2, CF@CS@CNT3, and CF@CS@CNT4, respectively.

For the selection of CNT concentrations, based on previous research, we found that the concentration of CNTs significantly affected the mechanical properties of composites. Mechanistically, CNTs have the following roles in improving the properties of carbon fiber composites IFSS: (1) CNTs will increase the surface roughness and specific surface area of CFs, improving the mechanical locking and physical entanglement with resin; (2) CNTs can guide the crack deflection away from the CF surface, relieving the stress concentration; (3) the carboxyl group on the CNTs can react with the epoxy group in resin and strengthen the adhesion between CFs and resin by chemical bonding. Therefore, we selected a series of CNT concentrations to evaluate the effect of CNT content on improving interface bonding performance. The results showed when the CNT concentration was low, the number of CNTs grafted on the CF surface was too small to significantly exert the mechanical locking cooperation between CNTs and the resin matrix. When the CNT concentration is high, the CNT layer grafted on the CFs surface will become thicker, leading to the degradation of IFSS performance. Therefore, based on the above considerations, four concentrations of 0.025%, 0.05%, 0.1%, and 0.15% were used to measure the effect of concentration on the interfacial properties of fibers and resins.

For comparison, CF-CS, where CS was physically grafted onto CFs, was also prepared through the following steps: 0.1 g CF-COOH was added to a 100 mL CS/DMF (0.1 wt.%) solution for 5 min, and then removed and washed by using deionized water, followed by drying in an oven at 60 °C for 24 h. In addition, CF@CS-CNT2 fibers, where CNTs were physically grafted onto CF@CS, were prepared by the following steps: 0.1 g CF@CS was added to 100 mL CNTs/DMF dispersion (0.05 wt.%) for 5 min, and then removed and washed by using deionized water, followed by drying in an oven at 60 °C for 24 h.

### 2.3. Characterization

**Surface characterizations.** The surface morphology of the samples was characterized by using scanning electron microscopy (SEM, Hitachi S4800, Hitachi, Japan) and electron microscopy (TEM, HT7700, Hitachi, Japan). The extent of defects in CNTs was characterized using laser confocal Raman spectroscopy (LABRAM HR Horriba-Jobin Yvon, Japan), and the extent of defects was evaluated by I_D_/I_G_, where I_D_ represents the intensity value of defects in C-atom crystals, and I_G_ represents the intensity value of in-plane stretching vibrations of C-atom sp^2^ hybridization [55]. The fiber surface elemental composition was determined by X-ray photoelectron spectroscopy (XPS), and the molecular structure and chemical composition of the fibers were characterized by using Fourier infrared spectroscopy (Nicolet 6700, Nicolet, Waltham, MA, USA), where the scanning wavenumber was in the range of 4000~400 cm^−1^.

**Figure 1:**
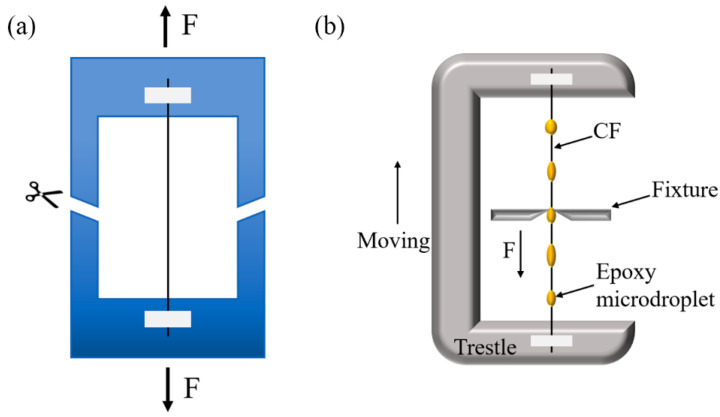
Schematics of mechanical testing: (**a**) carbon fiber monofilament tensile testing, (**b**) microdroplet testing.

**Tensile testing of single carbon fibers.** The monofilament tensile tests were performed on a universal materials tester (nano UTM 150, 500 mN, Agilent Technologies Co., Ltd., Santa Clara, CA, USA) according to the standard of ASTM D3822/D3822M-14 (2020), as shown in Figure 1a. The gauge length was set as 10 mm, and the monofilaments were stretched at a rate of 0.5 mm/s. The tensile strength of each monofilament was calculated as $\sigma =F_{b}/(\pi d^{2})$, where $F_{b}$ is the fracture load and *d* is the diameter of the carbon fiber. For every sample, over 50 tests were performed. The cumulative probability of failure of a fiber at a stress level equal to or less than σ is given as *P*$\left(\sigma \right)=1-exp\left[{-\left(\frac{\sigma }{\sigma_{0}}\right)}^{m}\right]$, where $\sigma_{0}$ is the scale parameter at the corresponding test span, and *m* is the shape parameter of the fiber, also known as the Weibull parameter [56]. A larger value of *m* indicates a smaller dispersion of monofilament strength, and vice versa.

**Interfacial shear strength testing.** Interfacial shear strength (IFSS) has been widely investigated by microbond (microdroplet) and fragmentation tests. In this study, the interfacial shear behaviors between CFs and the resin matrix were investigated using a microdroplet tester (MODEL HM410, Tohei Sangyo Co., Ltd., Tokyo, Japan). The specimens were prepared through the following procedures: (1) a fiber monofilament was fixed in a metal frame, (2) the mixture of TED-85 resin and curing agent (5/2) was scraped on the fiber surface to form resin microdroplets on the fiber, and (3) these droplets were then cured at 90 °C for 1 h, 120 °C for 2 h, and 150 °C for 3 h. During the test, fibers were drawn at a speed of 0.12 mm/min, and microdroplets were prevented from moving by a pair of rigid blades, as schematically illustrated in Figure 1b. The force variation during the test was recorded. The IFSS was calculated by equation $IFSS=F_{max}/(\pi dl)$, where $F_{max}$ is the maximum shear force between the fiber and solid resin microdroplet, *d* is the diameter of a single carbon fiber, and *l* is the diameter of the microdroplet.

## 3. Results and Discussion

### 3.1. Microstructures of the CFs Surface

**Figure 2:**
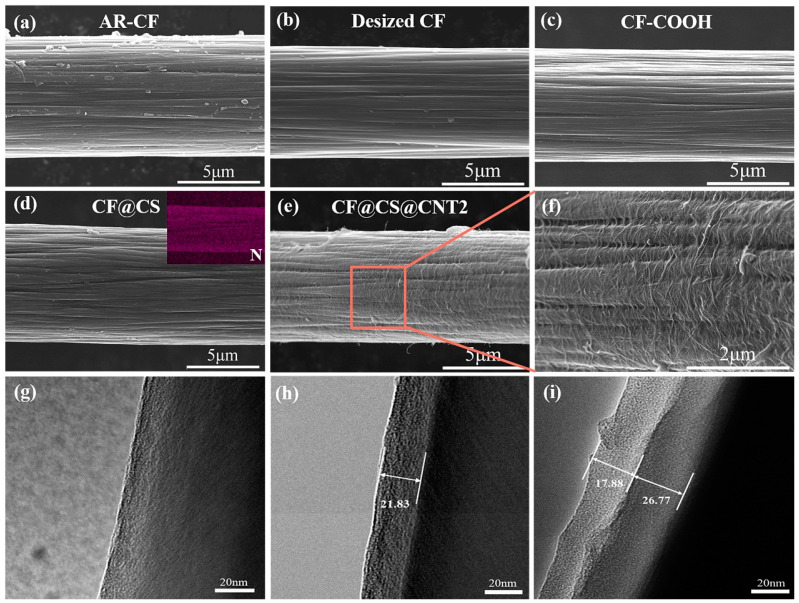
SEM images of (**a**) AR-CF, (**b**) desized CF, (**c**) CF -COOH, (**d**) CF@CS, and (**e**,**f**) CF@CS@CNT2; TEM images of (**g**) desized CF, (**h**) CF@CS, and (**i**) CF@CS@CNT2.

Figure 2a–f show the SEM images of the surface morphology of CFs before and after surface treatment. The longitudinal grooves were observed on the surfaces of all fibers. Some microparticles were observed on the surface of AR-CF (Figure 2a), while the surfaces of the desized CF (Figure 2b), CF-COOH (Figure 2c), and CF@CS (Figure 2d) did not show the microparticles. In addition, the EDS spectrum of the surface of CF@CS, as shown in Figure S1 and the inset of Figure 2d, show that N was homogeneously distributed on the surface of CF@CS, indicating that CS was uniformly coated on the CFs surface. Figure 2e,f show the SEM images of CF@CS@CNT2, suggesting that CNTs were evenly distributed on the fiber surface. The surfaces of desized CF, CF@CS, and CF@CS@CNT2 were further investigated through TEM analyses, as shown in Figure 2g, 2h, and 2i, respectively. For CF@CS (Figure 2h), the CF surface was uniformly coated by a CS layer with a thickness of approximately 21.83 nm. For CF@CS@CNT2 (Figure 2i), there were two distinguishable coating layers on the CFs surfaces, and the thicknesses of the CS and CNT layers were approximately 17.88 nm and 26.77 nm, respectively. The CNTs were clustered together on the surface of the CF, forming a “network-like” structure (Figure S2). The surface morphologies of other CF@CS@CNT samples were also investigated and are shown in Figure S3. As the CNT concentration increased, the CNTs “entangled” more tightly on the CF@CS surface (Figure S3a–d), and some agglomerates appeared on the CF@CS@CNT4 surface when the CNT concentration reached 0.015 wt.% (Figure S3d).

### 3.2. Characterization of the Chemical Composition of the CFs Surface

**Figure 3:**
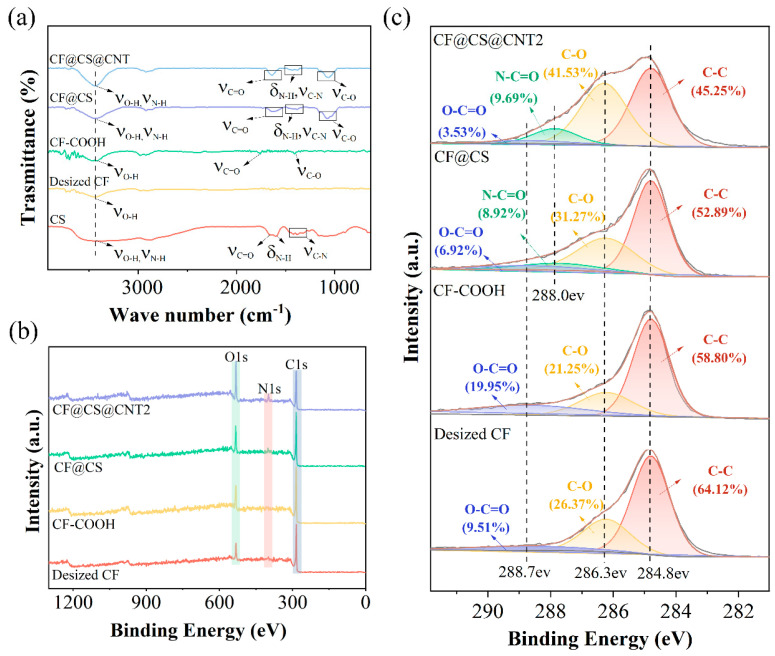
Surface chemical properties of CFs. (**a**) FT-IR spectra of each sample and (**b**) full scan XPS spectra. (**c**) C 1s spectra of different samples.

Figure 3a shows the IR spectra of all samples. For the desized CF sample, the broadband centered at 3440 cm^−1^ corresponded to the stretching vibration of O-H. Compared with desized CF, CF-COOH showed another two single peaks at 1409 and 1737 cm^−1^, which corresponded to the stretching vibration of C-O and C=O bonds, indicating the successful grafting of the carboxyl functional groups. The peaks located at 3440, 1625, 1510, and 1373 cm^−1^ in the spectrum of the CS were aligned to the stretching vibration of O-H, N-H, amide I band, bending vibration of N-H, and amide III band. The CF@CS showed the characteristic peaks of CS, suggesting the successful coating of CS on CF-COOH by the aminoacylation reaction. When CF@CS was further modified with CNTs, the peaks of the amide I band (1625 cm^−1^) and amide III band (1373 cm^−1^) were stronger, indicating that the carboxylated CNTs reacted with -NH_2_ from CS grafted on the surface of the CFs [57].

XPS spectroscopy was conducted to analyze the chemical components of the samples to evaluate the CF modification process. Figure 3b presents the full survey of the XPS spectrum of all samples. All samples showed a C 1s peak at 284.8 eV and an O 1s peak at 532.1 eV. The N 1s peak at 400.3 eV appeared in the spectra of the CF@CS and CF@CS@CNT2 samples, which was consistent with the FT-IR results. The desized CFs surface contained oxygen because the commercial CFs required electrochemical oxidation prior to sizing to increase surface active sites.

The C 1s spectra of all samples are shown in Figure 3c. According to split-peak fitting, the C 1s peaks of the desized CF and CF-COOH can be split into three peaks, centered at 284.4, 286.3, and 288.7 eV, which correspond to the C-C bond, C-O bond, and the O-C=O bond, respectively. Compared to desized CF, the O-C=O peak of CF-COOH increased significantly from 9.51% to 19.95% due to the increased oxygen content by the carboxylation treatment, which matched the element relative atom percentages in Table 1. In the presence of CS, the CF@CS and CF@CS@CNT2 samples can be split into four peaks, and a new peak at 288.0 eV appeared, which corresponded to the amide group (NH-CO), proving the successful grafting of CS. The CF@CS@CNT2 sample showed a stronger N-C≡O peak due to the greater aminoacylation reaction of CNTs and CS. The XPS spectrum and FT-IR results demonstrated the successful construction of multilayer interfacial structures.

Raman spectra and I_D_/I_G_ values of all samples are shown in Figure S4. The I_D_/I_G_ values for desized CF, CF-COOH, CF@CS, and CF@CS@CNT2 are 0.96, 0.99, 1.03, and 0.81, respectively. For carbon materials, there are two prominent peaks in their Raman spectra. The G band located at approximately 1580 cm^−1^ corresponds to graphitic enplane vibrations with E_2g_ symmetry. The D band at approximately 1360 cm^−1^ is attributed to the presence of defects and disorder, such as the presence of in-plane substitutional heteroatoms, grain boundaries, and aliphatic chains [20]. The I_D_/I_G_ values are influenced by the structure and proportional to the degree of defects on the surface of the carbon fibers. As the oxidation proceeded, the carbon fiber surfaces became more defective, resulting in an increase in the I_D_/I_G_ values. Due to its disorder, CS further increased the I_D_ value when it was wrapped around CFs. In addition, when CNTs were wrapped around the carbon fiber surface, the I_G_ value increased while the I_D_/I_G_ value decreased because of the highly graphitized structure of the CNTs.

**Figure 4:**
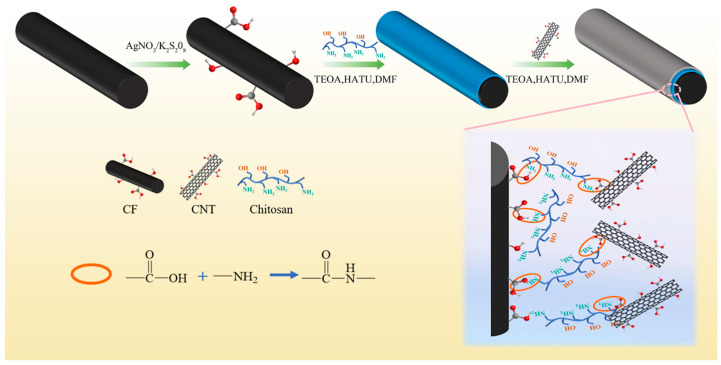
Synthesis schematic of CF@CS@CNT.

In this work, we first grafted CS onto the CFs surface by an amination reaction, and then chemically grafted CNTs onto the CF@CS surface by taking full advantage of the multi-amino group in the CS molecular structure to form a “layer-by-layer” multi-scale rigid–soft structure (Figure 4). In particular, the high-modulus CNTs acted as a strong barrier to deflect the crack path to consume the stress, while the soft phase CS absorbed the stress by deformation. Thus, an interface combining the advantages of soft and rigid phases will enhance the potential energy absorption of CFRP.

### 3.3. Interfacial Properties

**Figure 5:**
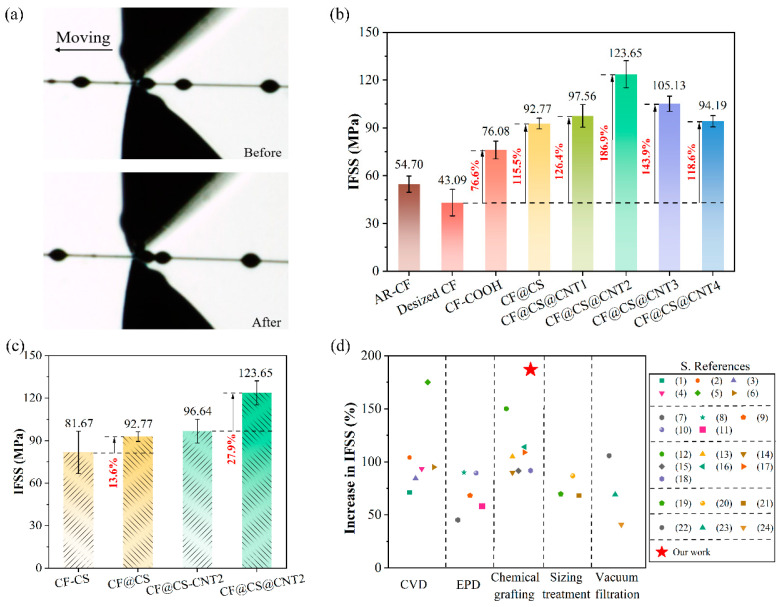
Interfacial properties of different samples. (**a**) Diagram of the testing process for IFSS; (**b**,**c**) IFSS values for different fibers; (**d**) comparison of IFSS performance with other carbon nanotube-modified carbon fiber methods (References [1–24] are cited in Supplementary Materials S6).

The interfacial shear performance of CFs was investigated through microdroplet tests. Figure 5a shows the optical images of the specimen before (upper) and after (lower) a test, where a droplet was forced to debond from and move along the fiber surface by two blades. The IFSS of different kinds of CFs are summarized and compared in Figure 5b. The raw data as well as typical stress–strain curves can be found in Figure S5, in the supplementary materials. The IFSS of the AR-CF was approximately 54.70 MPa, and it decreased to 43.09 MPa (21.2% lower) after the sizing materials were removed from the fiber surface. The IFSS was effectively enhanced by the surface modification developed in this study, and the IFSS of CF-COOH was approximately 76.08 MPa. After chemically grafting CS onto CF-COOH, the IFSS was further increased to approximately 92.77 MPa, which was 69.6% and 115.5% higher than those of AR-CF and desized fibers, respectively. For comparison, the IFSS of CF-CS, where CS was physically coated onto the CF-COOH surface, was approximately 81.67 MPa, and thus the IFSS of CF@CS was approximately 13.6% higher than that of CF-CS (Figure 5c), suggesting that the chemical grafting was more desirable for strengthening the interfacial shear performance of CFs.

The chemically grafting CNTs onto CF@CS further enhanced the IFSS, as shown in Figure 5b. The IFSS increased first with the CNT content, reaching the highest value of 123.65 MPa (CF@CS@CNT2), which was 126.1% and 187.0% higher than that of AR-CF and desized CF, respectively, and then decreased with further increases in CNT content.

For comparison, the IFSS of CF@CS-CNT2, where CNT2 was physically coated on the CF@CS surface, was approximately 99.64 MPa, and the IFSS of CF@CS@CNT2 was approximately 27.9% higher than that of CF@CS-CNT2 (Figure 5c), which again indicated that chemical grafting was more desirable for strengthening the interfacial shear performance of CFs. When increasing the CNT concentration, the IFSS performance showed a trend of increasing and then decreasing.

**Figure 6:**
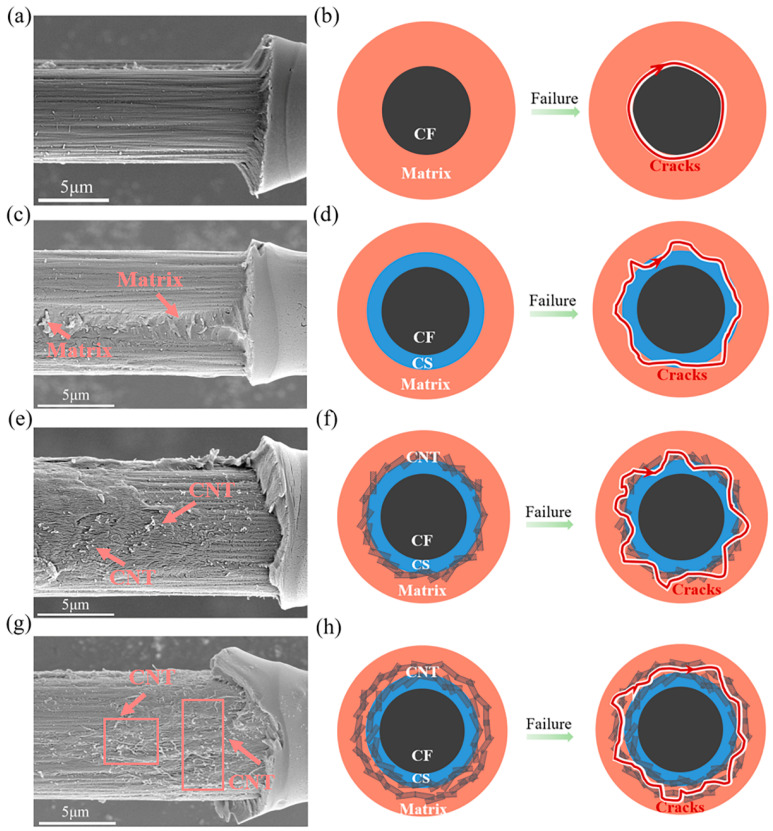
Surface of de-bonded carbon fibers and schematics of their failure modes: (**a**,**b**) desized CF, (**c**,**d**) CF@CS, (**e**,**f**) CF@CS@CNT2, and (**g**,**h**) CF@CS@CNT3.

For a better understanding of the roles of CS and CNTs on the interfacial shear behavior, the specimens after the microdroplet tests were observed by using SEM. For the desized CF, as shown in Figure 6a, the surface grooves can be well identified, and the fiber surface was mostly clear of resin materials. This indicated that the interfacial bonding between desized CF and resin was undesirable, and the failure mainly took place at the fiber surface, as illustrated in Figure 6b.

Figure 6c shows the microscopic structures of the CF@CS fiber after microdroplet testing. The grooves on the fiber surface were less obvious and the surface was covered by a layer of resin materials. As mentioned earlier, CS can form covalent bonds with both CF-COOH and EP; thus it established stronger bonding between these two materials. During the microdroplet testing, instead of failure along the CFs surface, failure was prone to take place within the EP near the CS/EP interface once the shear stress was higher than the shear strength of the EP, as illustrated in Figure 6d.

Figure 6e shows the surface of a debonded CF@CS@CNT2 fiber. The surface was relatively rough, and some CNTs were pulled out from the resin. CNTs that were chemically coated onto fibers played several possible roles in the IFSS. First, the separation of CNTs from CFs required relatively high strength due to the strong chemical bonding between CFs and CS and CNTs. On the other hand, the interlocking between CFs and the matrix enhanced the infiltration of CNTs into the matrix when the liquid matrix wrapped onto CFs, and the epoxy monomer inter-diffused through the CNT-grafted network and interacted chemically with the carboxyl groups of the CNTs and the amine groups of unreacted melamine. Thus, a larger strength was required to pull the CNTs out of the resin. Moreover, the increase in surface roughness caused by the increasing CNT concentration may further increase the interfacial strength. Finally, the cracks propagated in the resin during microdroplet testing were deflected when encountering CNTs, as illustrated in Figure 6f.

The surfaces of the debonded CF@CS@CNT3 were also characterized. As shown in Figure 6g, a layer of CNT/EP composite on the fiber surface was detected, where a large quantity of CNTs was found. Compared with CF@CS@CNT2, CF@CS@CNT3 had more CNTs on its surface, as shown in Figure S3c, which resulted from the higher concentration of CNTs in CNT dispersions adopted for making CF@CS@CNT3. However, due to the “overcovering” of CNTs, on one hand, CNTs that contacted intimately with CS can form strong covalent bonds between them, while the remaining CNTs only interacted with each other through van der Waals interactions. Due to the weak van der Waals interactions between the CNTs, interfacial defects may appear inside the CNTs, resulting in lower IFSS values. On the other hand, when the CNT films became thicker, CNTs would agglomerate on the surface, which may cause local stress concentration and reduce energy dissipation [42]. The narrow gap among CNTs can restrict the influx of resin to the CFs surface [58,59] and result in a CNT/EP composite layer with weak shear resistance. Thus, it is plausible to speculate that during microdroplet testing, shear-induced failure occurred within the coated CNT/EP composite layers, as illustrated in Figure 6h.

Figure 5d summarizes some of the reported enhancement of IFSS after coating CNTs onto carbon fibers by using methods such as CVD, EPD, sizing treatment, vacuum filtration, and chemical grafting. Among them, this study obtained the highest IFSS enhancement, which was approximately 186.9%, indicating that the proposed strategy was more effective in strengthening the IFSS of carbon fibers.

### 3.4. Single-Fiber Tensile Strength

**Figure 7:**
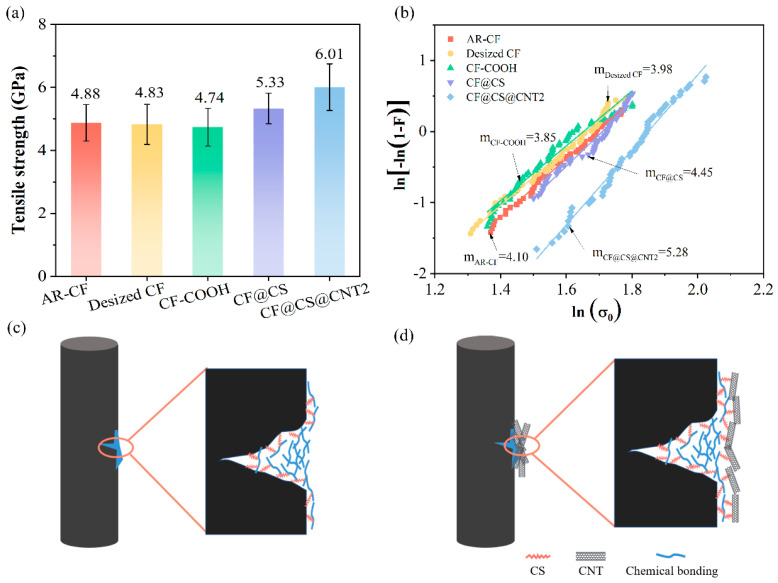
The tensile performance of different carbon fibers: (**a**) single-fiber tensile strength and (**b**) Weibull distribution curves of different carbon fibers, and the schematics of the strengthening mechanisms of (**c**) CF@CS and (**d**) CF@CS@CNT2.

It is well recognized that the mechanical properties of carbon fibers play a significant role in determining the load-carrying capability of composites. It is necessary to examine the effects of surface treatment on the mechanical properties of single carbon fibers. Some previous studies have reported that carbon fibers can be strengthened after surface coating [33,38,60,61]. In this study, the single-fiber tensile testing (SFTT) of various carbon fibers before and after surface treatment was performed. Figure 7a summarizes the average tensile strength of AR-CF, desized CF, CF-COOH, CF@CS, and CF@CS@CNT2, which was 4.88 (±0.58), 4.83 (±0.64), 4.74 (±0.60), 5.33 (±0.49), and 6.01 (±0.74) GPa, respectively. This indicated that the carbon fibers were marginally degraded after the desizing and oxidation processing, while the further coating of CS and CNTs effectively strengthened the fibers. Specifically, the tensile strengths of CF@CS and CF@CS@CNT2 were approximately 12.45% and 26.79% higher than that of CF-COOH. Griffith’s microcrack theory [62] suggested that when stretched, CFs experience stress concentration at the defects, which facilitated the initiation and propagation of cracks. After coating with CS, it was assumed that CS would fill in the voids on the fiber surface and form strong bonds with the surrounding graphites, as illustrated in Figure 7c. This would alleviate the stress concentration at these locations during the fiber stretching and thus delay the crack propagation. In addition, the CS coated on the other part of the fiber surface also covalently bonded with CFs and formed a tough layer on the fiber surface, which would potentially delay the initiation of surface cracks upon loading. Both of these two issues effectively enhance the tensile strength of the fiber (Figure 7c). Similarly, after coating CNTs onto the CS layer, the covalent bonds between CS and CNTs would make the surface coating layer stronger and tougher, thus further enhancing the tensile strength of the fibers further (Figure 7d).

As the CNT concentrations increased, the tensile properties of CF@CS@CNT improved slightly, as shown in Table S1. The experimental results showed that the monofilament tensile properties of CFs were improved slightly with the increase in CNT concentrations, because CS and CNTs can compensate for fiber surface defects and increase the radius of curvature of the crack front during crack propagates, which effectively prevented stress concentration [38,63]. In addition, the microcrack bridging effect of the homogeneous distribution of CNTs in the surrounding areas of the CFs surface also contributed to the enhancement of tensile properties [64]. In this work, various CNT concentrations were used to investigate the effect of CNTs on IFSS properties, and the monofilament tensile test was only used to prove that the method we proposed was not detrimental to the mechanical properties of the fibers. Therefore, in this work, we chose IFSS performance as the main consideration factor to determine the effect of CF surface modification, and CF@CS@CNT2, which showed the highest IFSS performance, was selected to be studied in detail.

The discreteness of the tensile strength of different kinds of CFs was also evaluated through the Weibull analyses. As shown in Figure 7b, the shape parameters of the fiber, *m*, for AR-CF, desized CF, CF-COOH, CF@CS, and CF@CS@CNT2 were 4.10, 3.98, 3.85, 4.45, and 5.28, respectively. Considering that a larger value of *m* indicates a smaller dispersion of monofilament strength, it was concluded that the coating of CS and CNTs could reduce the discreteness of the fiber strength in addition to strengthening the fiber. Although the exact underlying mechanisms are still unclear, it is expected that the tougher layer coated on the fiber surface would result in a relief of stress concentration in the fibers and make each kind of fiber break more uniformly.

## 4. Conclusions

This work presented the establishment of a multistage gradient modulus CS@CNT “rigid-soft” interphase to improve the interfacial strength of CF/epoxy composites through an efficient and non-destructive two-step chemical grafting method. The IFSS values of CF@CS and CF@CS@CNT2 were 115.5% and 186.9% higher than that of desized CF. The “rigid-soft” interphase increased the CF surface roughness, which enhanced the mechanical locking. In addition, the covalently bonded nanomaterials were found to have better interfacial strengthening efficiency than the non-covalently bonded nanomaterials. Furthermore, carbon fibers were found to be strengthened after coating with CS and CNTs. The tensile strength of CF@CS and CF@CS@CNT2 was 12.45% and 26.79% higher than that of non-coated CFs. Therefore, the study of establishing a “rigid-soft” gradient modulus interface layer by simple chemical bond bridging would provide a valuable reference for future interface optimization to obtain high-performance CF composites.

## Figures and Tables

**Table 1:** Relative percentages of XPS fractions of different samples.

Samples	C/N/O (%)	C1s (%)			
		C-C	C-O	O-C=O	N-C=O
**desized CF**	85.5/2.5/12.0	64.1	26.4	9.5	—
**CF-COOH**	83.7/3.2/13.1	58.8	21.3	19.9	—
**CF@CS**	82.3/3.3/14.4	52.9	31.3	6.9	8.9
**CF@CS@CNT2**	69.2/6.6/24.2	45.3	41.5	9.7	3.5

## Data Availability

The data that supports the findings above can be requested from corresponding author.

## Supplementary Materials

The following supporting information can be downloaded at: https://www.mdpi.com/article/10.3390/polym15092147/s1, Figure S1. EDS diagrams of CF@CS; Figure S2. Large magnification TEM image of the CNT layer in CF@CS@CNT2; Figure S3. SEM images: (a) CF@CS@CNT1, (b) CF@CS@CNT2, (c) CF@CS@CNT3, (d) CF@CS@CNT4; Figure S4. Raman images of different samples; Figure S5. IFSS specific data; Figure S6. IFSS performance comparison; Table S1. Comparison of the performance of different samples.
