# Supplementary material for: Enhancing the Interfacial Shear Strength and Tensile Strength of Carbon Fibers through Chemical Grafting of Chitosan and Carbon Nanotubes
Source: Polymers (Basel). 2023 Apr 30;15(9):2147. doi: 10.3390/polym15092147 (PMC10181124; doi:10.3390/polym15092147)
Supplement: Supplementary file 1 [file polymers-15-02147-s001.zip › polymers-2308290-supplementary.pdf]

polymers

MDPI

Supplementary

Enhancing the Interfacial Shear Strength and Tensile Strength of Carbon Fibers through Chemical Grafting of Chitosan and Carbon Nanotubes

Jingyue Xiao [1,2], Huigai Li [3], Munan Lu [2], Yuqiong Wang [2], Jin Jiang [2], Wengang Yang [2], Shuxuan Qu [2,*] and Weibang Lu [2,4,*]

1 School of Nano-Tech and Nano-Bionics, University of Science and Technology of China, Hefei 230026, China
2 Division of Advanced Nanomaterials and Innovation Center for Advanced Nanocomposites, Suzhou Institute of Nano-Tech and Nano-Bionics, Chinese Academy of Sciences, Suzhou 215123, China
3 College of Textile and Clothing Engineering, Soochow University, Suzhou 215021, China
4 Division of Nanomaterials and Jiangxi Key Lab of Carbonene Materials, Jiangxi Institute of Nanotechnology, Nanchang 330200, China
* Correspondence: sxqu2013@sinano.ac.cn (S.Q.); wblv2013@sinano.ac.cn (W.L.)

EDS analysis of CF@CS surface elements:

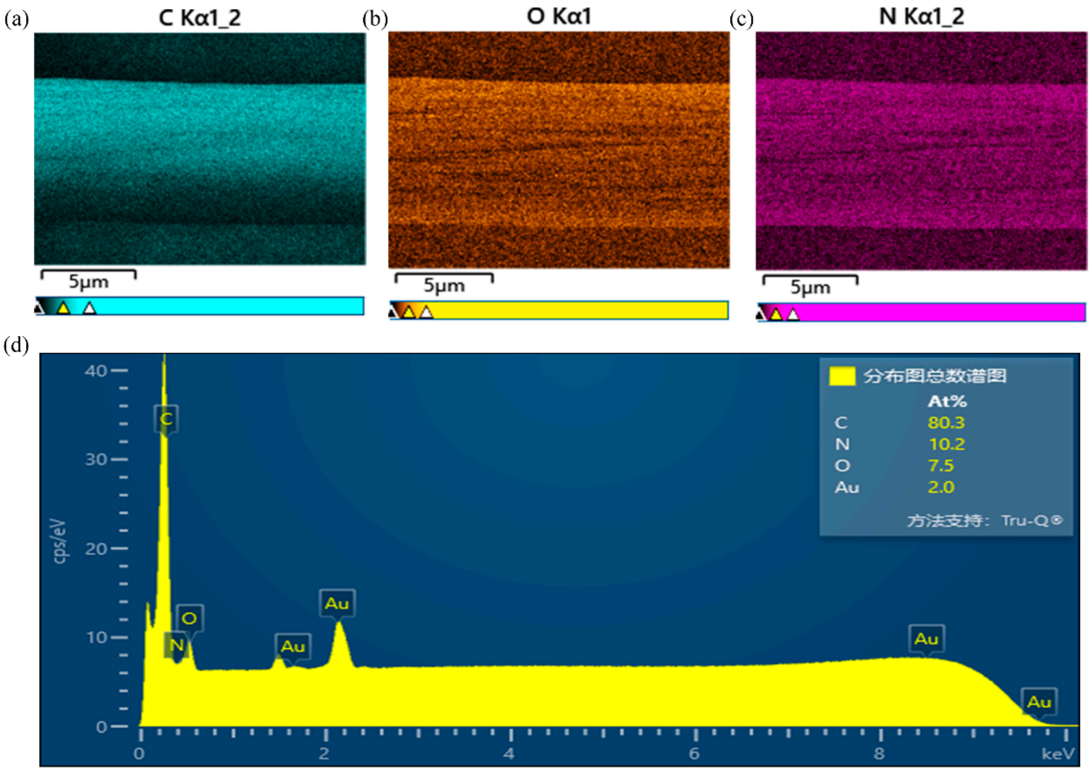

Figure S1. EDS diagrams of CF@CS: (a) distribution image of C, (b) distribution image of O, (c) distribution image of N, (d) total spectrum of distribution image.

Large magnification TEM image of the CNT layer in CF@CS@CNT2: The CNTs were clustered together on the surface of CF, forming a "network-like" structure with no obvious orientation.

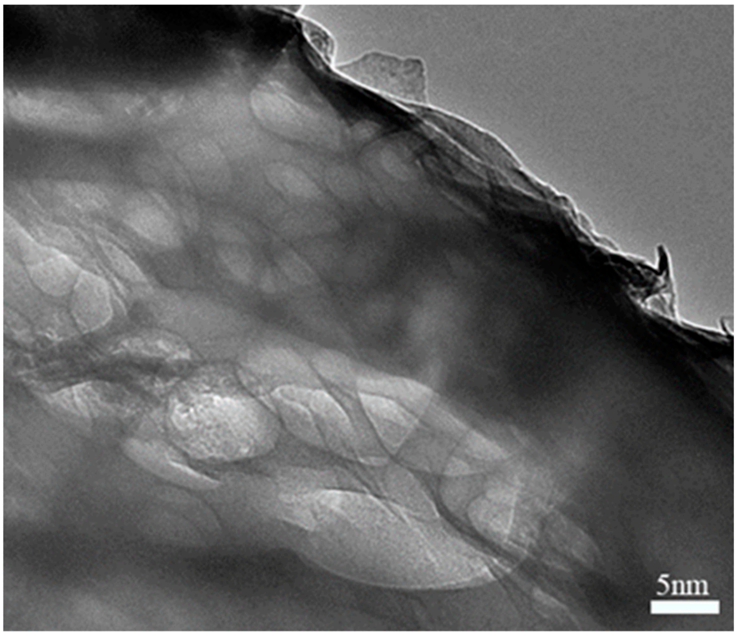

Figure S2. Large magnification TEM image of the CNT layer in CF@CS@CNT2.

SEM images of different contents of CNTs: (a) CF@CS@CNT1 for 0.025wt.% of CNTs (b) CF@CS@CNT1 for 0.05wt.% of CNTs (c) CF@CS@CNT1 for 0.1wt.% of CNTs (d) CF@CS@CNT1 for 0.15wt.% of CNTs

The morphological changes were observed when the samples were grafted with different concentrations of CNTs, as the CFs surface roughness increased significantly. When the CNTs concentration was 0.025 wt.% since the amino group of CS could react chemically with the carboxyl groups of different parts of the CNTs, resulting in an inverted distribution of CNTs on the surface of CF (Fig. S2a). However, due to the low CNTs concentration, the CNTs were randomly dotted and unevenly distributed on the CFs surface. When the CNT concentration increased further, the CNTs were more and more densely distributed on the CFs surface, and the distance between the tube bundles decreased, resulting in a uniform CNTs network layer (Fig. S2b). When the CNTs concentration was 0.1 wt.%, due to the strong van der Waals forces of the CNTs, the densely grafted CNTs showed little agglomeration on the CFs surface (Fig. S2c). Upon increasing the CNTs concentration to 0.15 wt.%, the bundles of CNTs became closer together and more obvious agglomeration occurred (Fig. S2d).

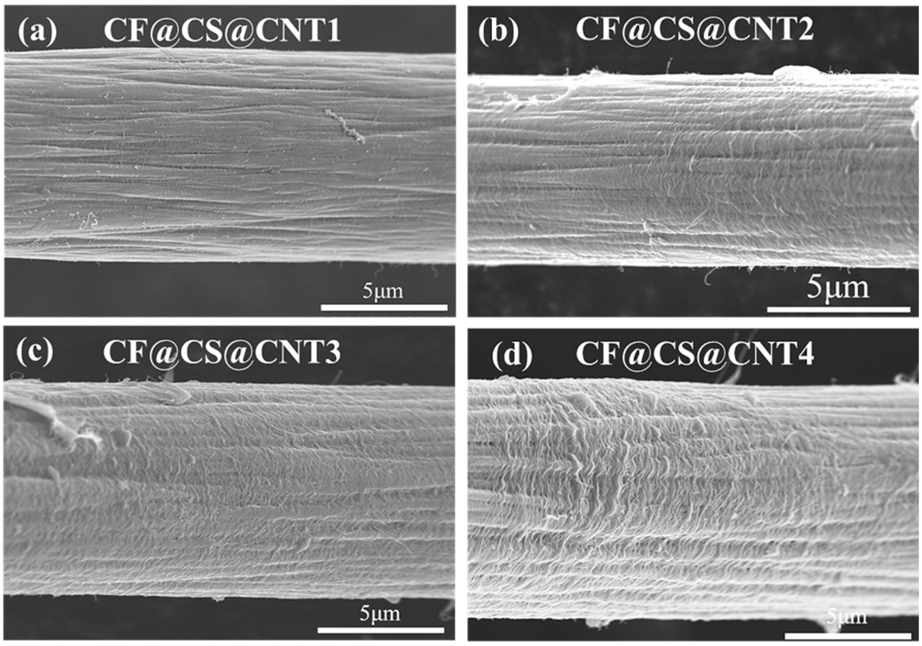

Figure S3. SEM images: (a) CF@CS@CNT1, (b) CF@CS@CNT2, (c) CF@CS@CNT3, (d) CF@CS@CNT4.

Raman images of different samples:

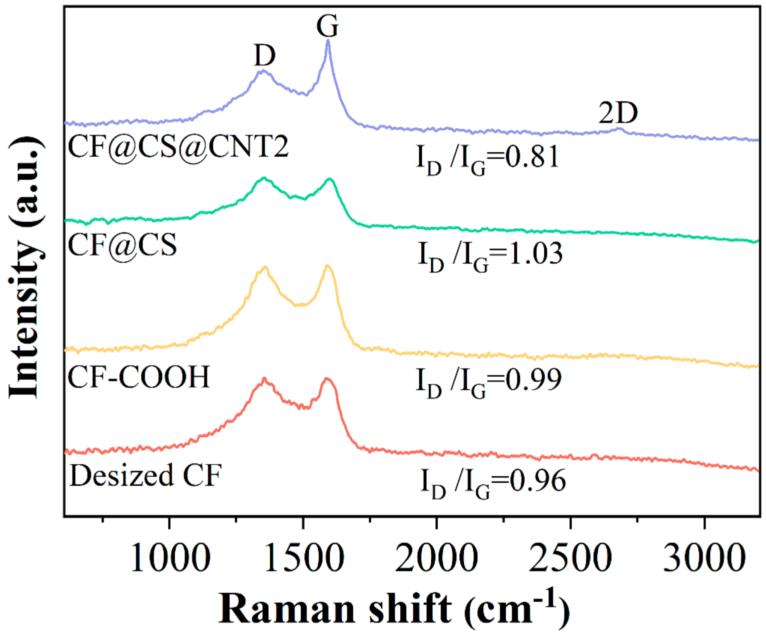

Figure S4. Raman images of different samples.

Specific data for each sample IFSS:

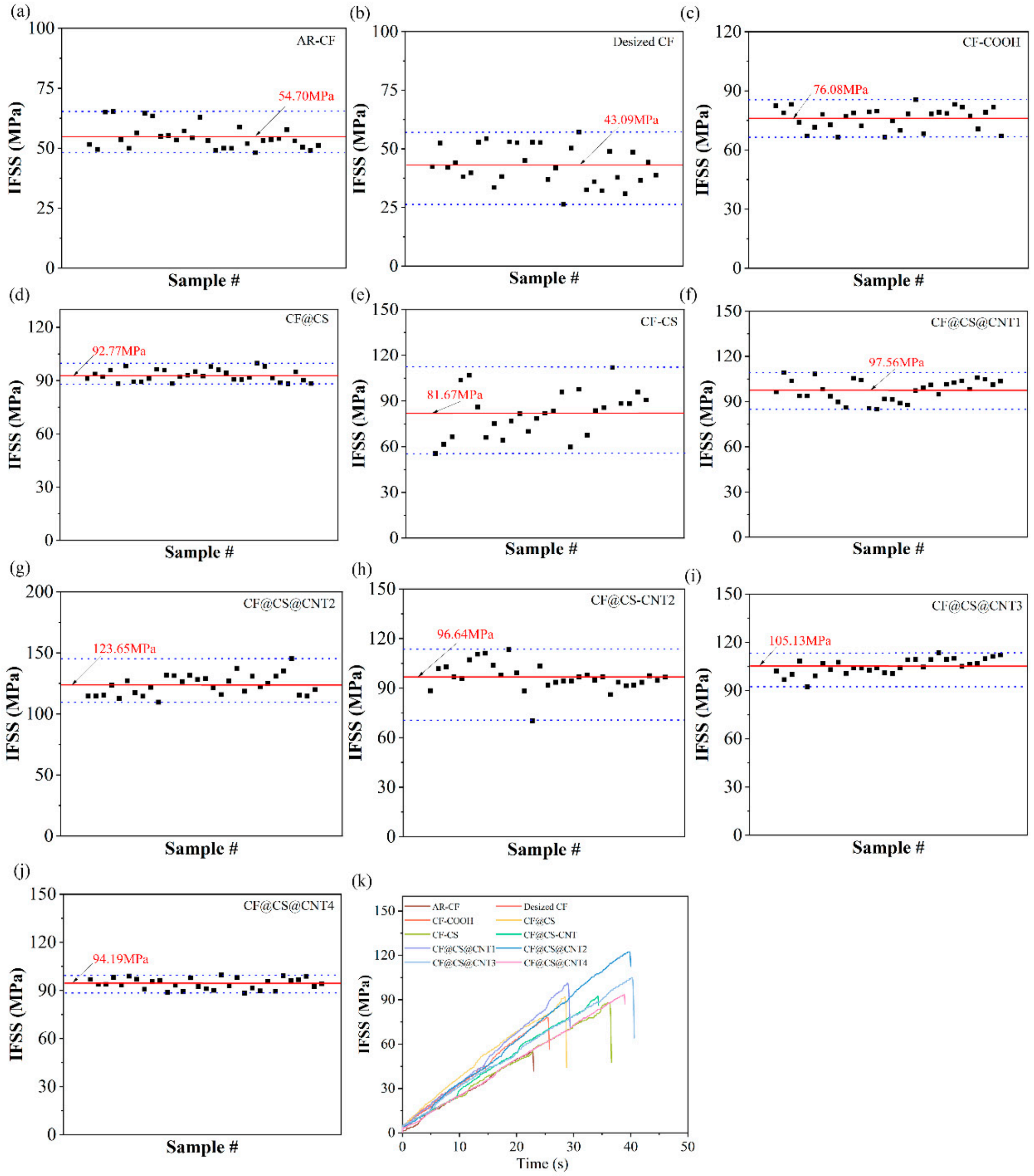

Figure S5. IFSS specific data: (a) AR-CF, (b) desized CF, (c) CF-COOH, (d) CF@CS, (e) CF-CS, (f) CF@CS@CNT1, (g) CF@CS@CNT2, (h) CF@CS-CNT2, (i) CF@CS@CNT3, (j) CF@CS@CNT4, (k) The typical stress-strain curves for IFSS.

Comparison of IFSS performance with other literature and references:

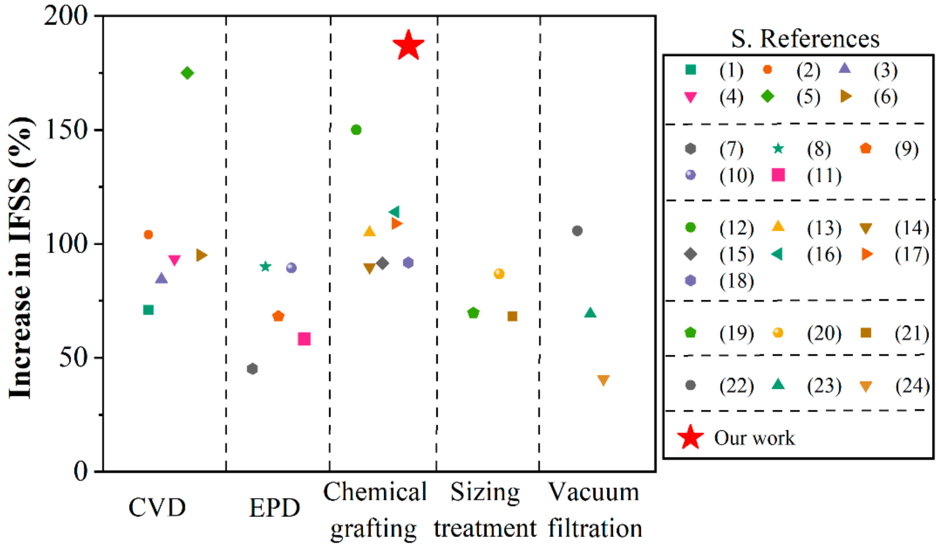

Figure S6. IFSS performance comparison.

Summary table of the performance of the samples with different CNT concentrations:

Table S1. Comparison of the performance of different samples.

Sample	CNT concentrations (wt.%)	IFSS (MPa)	Tensile strength (GPa)
CF@CS@CNT1	0.025	97.56	5.87 (±0.94)
CF@CS@CNT2	0.05	123.65	6.01 (±0.74)
CF@CS@CNT3	0.1	105.13	6.04 (±0.87)
CF@CS@CNT4	0.15	94.19	6.11 (±0.81)

As CNTs concentrations increased, the tensile properties of CF@CS@CNT improved slightly, as shown in Tab. S1. In this work, various CNTs concentrations were used to investigate the effect of CNTs on IFSS properties, and the monofilament tensile test was only used to prove that the method we proposed was not detrimental to the mechanical properties of the fibers. Therefore, in this work, we chose IFSS performance as the main consideration factor to determine the effect of CF surface modification, and CF@CS@CNT2, which showed the highest IFSS performance, was selected to be detailed studied. For CF@CS@CNT2, this apparent increase can be attributed to three aspects: (1) The increase of CNT concentration will increase the surface roughness of CFs and enhance the mechanical locking between fiber and resin. (2) The functionalized CNTs can react chemically with the resin to enhance the strength of the epoxy resin at the interface; the connection between CF and CS, CS and CNTs all rely on strong covalent bonds, and the CNTs can not be peeled off easily from the interface when subjected to shear forces. Therefore, a higher force is needed to pull CNTs out of the resin. (3) The cracks propagating in the resin will be deflected away from the CFs surface when they encounter CNTs.

However, the IFSS of CF@CS@CNT3 decreased slightly from 123.65 MPa to 105.13 MPa due to the "over-covering" of CNTs. On the one hand, too much CNTs would increase the thickness of the interfacial layer, which is not conducive to stress transfer; on the other hand, in the over-thick CNTs layer, only some of the CNTs in close contact with CS could form strong covalent bonds, while the rest of CNTs can only interact with each other through van der Waals interaction. Due to the weak van der Waals interaction between CNTs, interfacial defects may appear inside the CNTs, leading to a decrease in

IFSS values. In addition, narrow gaps between CNTs can restrict the flow of resin to the CF's surface, resulting in poor interfacial adhesion between CFs and epoxy resin[25,26].

S. References

1. Anthony, D.B.; Sui, X.; Kellersztein, I.; De Luca, H.G.; White, E.R.; Wagner, H.D.; Greenhalgh, E.S.; Bismarck, A.; Shaffer, M.S.P. Continuous carbon nanotube synthesis on charged carbon fibers. Composites Part A: Applied Science and Manufacturing **2018**, 112, 525-538, doi:https://doi.org/10.1016/j.compositesa.2018.05.027.
2. Lutz, V.; Duchet-Rumeau, J.; Godin, N.; Smail, F.; Lortie, F.; Gérard, J.F. Ex-PAN carbon fibers vs carbon nanotubes fibers: From conventional epoxy based composites to multiscale composites. European Polymer Journal **2018**, 106, 9-18, doi:https://doi.org/10.1016/j.eurpolymj.2018.06.023.
3. Zheng, L.; Wang, Y.; Qin, J.; Wang, X.; Lu, R.; Qu, C.; Wang, C. Scalable manufacturing of carbon nanotubes on continuous carbon fibers surface from chemical vapor deposition. Vacuum **2018**, 152, 84-90, doi:https://doi.org/10.1016/j.vacuum.2018.03.011.
4. Lee, G.; Sung, M.; Youk, J.H.; Lee, J.; Yu, W.-R. Improved tensile strength of carbon nanotube-grafted carbon fiber reinforced composites. Composite Structures **2019**, 220, 580-591, doi:https://doi.org/10.1016/j.compstruct.2019.04.037.
5. Lv, P.; Feng, Y.-y.; Zhang, P.; Chen, H.-m.; Zhao, N.; Feng, W. Increasing the interfacial strength in carbon fiber/epoxy composites by controlling the orientation and length of carbon nanotubes grown on the fibers. Carbon **2011**, 49, 4665-4673, doi:https://doi.org/10.1016/j.carbon.2011.06.064.
6. Liu, C.; Miao, L.; Zhao, R.; Yang, F.; Unnikrishnan, V.; Rana, R.; Deng, N.; Ghandehari Ferdowsi, M.R.; Chao, Q.; Kang, W.; et al. In-situ fabrication of multifunctional N-doped hybrid carbon nanotube@carbon fiber by recycling gaseous effluents of carbon fiber production. Carbon **2022**, 193, 368-380, doi:https://doi.org/10.1016/j.carbon.2022.03.046.
7. Zhao, Z.; Teng, K.; Li, N.; Li, X.; Xu, Z.; Chen, L.; Niu, J.; Fu, H.; Zhao, L.; Liu, Y. Mechanical, thermal and interfacial performances of carbon fiber reinforced composites flavored by carbon nanotube in matrix/interface. Composite Structures **2017**, 159, 761-772, doi:https://doi.org/10.1016/j.compstruct.2016.10.022.
8. Moaseri, E.; Karimi, M.; Maghrebi, M.; Baniadam, M. Fabrication of multi-walled carbon nanotube–carbon fiber hybrid material via electrophoretic deposition followed by pyrolysis process. Composites Part A: Applied Science and Manufacturing **2014**, 60, 8-14, doi:https://doi.org/10.1016/j.compositesa.2014.01.009.
9. Liu, Y.-T.; Song, H.-Y.; Yao, T.-T.; Zhang, W.-S.; Zhu, H.; Wu, G.-P. Effects of carbon nanotube length on interfacial properties of carbon fiber reinforced thermoplastic composites. Journal of Materials Science **2020**, 55, 15467-15480, doi:10.1007/s10853-020-05129-w.
10. Yao, X.; Gao, X.; Jiang, J.; Xu, C.; Deng, C.; Wang, J. Comparison of carbon nanotubes and graphene oxide coated carbon fiber for improving the interfacial properties of carbon fiber/epoxy composites. Composites Part B: Engineering **2018**, 132, 170-177, doi:https://doi.org/10.1016/j.compositesb.2017.09.012.
11. Li, L.; Liu, W.; Yang, F.; Jiao, W.; Hao, L.; Wang, R. Interfacial reinforcement of hybrid composite by electrophoretic deposition for vertically aligned carbon nanotubes on carbon fiber. Composites Science and Technology **2020**, 187, 107946, doi:https://doi.org/10.1016/j.compscitech.2019.107946.
12. Zhang, F.-H.; Wang, R.-G.; He, X.-D.; Wang, C.; Ren, L.-N. Interfacial shearing strength and reinforcing mechanisms of an epoxy composite reinforced using a carbon nanotube/carbon fiber hybrid. Journal of Materials Science **2009**, 44, 3574-3577, doi:10.1007/s10853-009-3484-x.
13. Zhao, F.; Huang, Y. Preparation and properties of polyhedral oligomeric silsesquioxane and carbon nanotube grafted carbon fiber hierarchical reinforcing structure. Journal of Materials Chemistry **2011**, 21, 2867-2870, doi:10.1039/C0JM03919E.
14. Xu, N.; Li, Y.; Zheng, T.; Xiao, L.; Liu, Y.; Chen, S.; Zhang, D. A mussel-inspired strategy for CNT/carbon fiber reinforced epoxy composite by hierarchical surface modification. Colloids and Surfaces A: Physicochemical and Engineering Aspects **2022**, 635, 128085, doi:https://doi.org/10.1016/j.colsurfa.2021.128085.
15. Zhang, C.; Zhang, X.; Luo, Y.; Liang, M.; Zou, H. Green and Nondestructive Method for Constructing Multiscale Carbon Fiber Reinforcement via Encapsulating Chitosan and Grafting Carbon Nanotubes. ACS Applied Nano Materials **2021**, 4, 13388-13397, doi:10.1021/acsanm.1c02890.
16. Liu, Y.-T.; Wu, G.-P.; Lu, C.-X. Grafting of carbon nanotubes onto carbon fiber surfaces by step-wise reduction of in-situ generated diazonium salts for enhancing carbon/epoxy interfaces. Materials Letters **2014**, 134, 75-79, doi:https://doi.org/10.1016/j.matlet.2014.07.053.
17. Chen, Q.; Peng, Q.; Zhao, X.; Sun, H.; Wang, S.; Zhu, Y.; Liu, Z.; Wang, C.; He, X. Grafting carbon nanotubes densely on carbon fibers by poly(propylene imine) for interfacial enhancement of carbon fiber composites. Carbon **2020**, 158, 704-710, doi:https://doi.org/10.1016/j.carbon.2019.11.043.
18. Lin, J.; Xu, P.; Wang, L.; Sun, Y.; Ge, X.; Li, G.; Yang, X. Multi-scale interphase construction of self-assembly naphthalenediimide/multi-wall carbon nanotube and enhanced interfacial properties of high-modulus carbon fiber composites. Composites Science and Technology **2019**, 184, 107855, doi:https://doi.org/10.1016/j.compscitech.2019.107855.
19. Liu, W.; Zhang, S.; Hao, L.; Yang, F.; Jiao, W.; Li, X.; Wang, R. Fabrication of carbon nanotubes/carbon fiber hybrid fiber in industrial scale by sizing process. Applied Surface Science **2013**, 284, 914-920, doi:https://doi.org/10.1016/j.apsusc.2013.08.045.

20. Xiong, S.; Zhao, Y.; Wang, Y.; Song, J.; Zhao, X.; Li, S. Enhanced interfacial properties of carbon fiber/epoxy composites by coating carbon nanotubes onto carbon fiber surface by one-step dipping method. Applied Surface Science **2021**, 546, 149135, doi:https://doi.org/10.1016/j.apsusc.2021.149135.
21. Liu, Y.-T.; Song, H.-Y.; Yao, T.-T.; Zhang, W.-S.; Zhu, H.; Wu, G.-P. Effects of carbon nanotube length on interfacial properties of carbon fiber reinforced thermoplastic composites. Journal of Materials Science **2020**, doi:10.1007/s10853-020-05129-w.
22. Wu, Q.; Bai, H.; Yang, X.; Zhu, J. Significantly increasing the interfacial adhesion of carbon fiber composites via constructing a synergistic hydrogen bonding network by vacuum filtration. Composites Part B: Engineering **2021**, 225, 109300, doi:https://doi.org/10.1016/j.compositesb.2021.109300.
23. Wu, Q.; Bai, H.; Deng, H.; Ye, Z.; Wang, Q.; Zhu, J. Intermittent carbon nanotube encapsulation of carbon fiber: A facile and efficient strategy to simultaneously strengthen and toughen interphase of composites. Composites Part B: Engineering **2022**, 235, 109785, doi:https://doi.org/10.1016/j.compositesb.2022.109785.
24. Wu, Q.; Bai, H.; Gao, A.; Zhu, J. High-density grafting of carbon nanotube/carbon nanofiber hybrid on carbon fiber surface by vacuum filtration for effective interfacial reinforcement of its epoxy composites. Composites Science and Technology **2022**, 225, 109522, doi:https://doi.org/10.1016/j.compscitech.2022.109522.
25. Zhao, M.; Meng, L.; Ma, L.; Ma, L.; Yang, X.; Huang, Y.; Ryu, J.E.; Shankar, A.; Li, T.; Yan, C.; et al. Layer-by-layer grafting CNTs onto carbon fibers surface for enhancing the interfacial properties of epoxy resin composites. Composites Science and Technology 2018, 154, 28-36, doi:10.1016/j.compscitech.2017.11.002.
26. Wang, C.; Li, Y.; Tong, L.; Song, Q.; Li, K.; Li, J.; Peng, Q.; He, X.; Wang, R.; Jiao, W.; et al. The role of grafting force and surface wettability in interfacial enhancement of carbon nanotube/carbon fiber hierarchical composites. Carbon 2014, 69, 239-246, doi:https://doi.org/10.1016/j.carbon.2013.12.020.
